# Symbiosis of executive and selective attention in working memory

**DOI:** 10.3389/fnhum.2014.00588

**Published:** 2014-08-08

**Authors:** André Vandierendonck

**Affiliations:** Department of Experimental Psychology, Ghent UniversityGhent, Belgium

**Keywords:** working memory, selective attention, executive control, executive attention, task-set switching

## Abstract

The notion of working memory (WM) was introduced to account for the usage of short-term memory resources by other cognitive tasks such as reasoning, mental arithmetic, language comprehension, and many others. This collaboration between memory and other cognitive tasks can only be achieved by a dedicated WM system that controls task coordination. To that end, WM models include executive control. Nevertheless, other attention control systems may be involved in coordination of memory and cognitive tasks calling on memory resources. The present paper briefly reviews the evidence concerning the role of selective attention in WM activities. A model is proposed in which selective attention control is directly linked to the executive control part of the WM system. The model assumes that apart from storage of declarative information, the system also includes an executive WM module that represents the current task set. Control processes are automatically triggered when particular conditions in these modules are met. As each task set represents the parameter settings and the actions needed to achieve the task goal, it will depend on the specific settings and actions whether selective attention control will have to be shared among the active tasks. Only when such sharing is required, task performance will be affected by the capacity limits of the control system involved.

## Introduction

It seems self-evident that the likelihood of successfully recalling a previously observed sequence of events will be larger when attention was than when it was not selectively oriented towards these events. Nevertheless, theories of working memory (WM) have been focusing on executive attention rather than on other kinds of attention like orienting attention, which is a main constituent of selective attention tasks. Furthermore, the evidence in support of a role for orienting or selective attention in WM has not been settled, and it remains an open question whether selective attention is indeed an indispensable aspect of WM. The presumption that selective attention plays a central role in WM is certainly tempting. The present article scrutinizes this hypothesis by reviewing the behavioral evidence regarding interactions between selective and executive attention in tasks taxing WM. This review will be restricted to studies relevant to the interaction of both kinds of attention and will therefore not be completely representative. In preview, the paper will show that both kinds of attention have a symbiotic relationship within WM.

At this point, it seems appropriate to provide some delineation of the attention categories involved in the present article. The term *selective attention* typically refers to the kind of processing involved in orienting attention towards a specific set of entities or representations while ignoring others. Such processing may be driven either by more automatic or by more top-down controlled processing (Schneider and Shiffrin, 1977; Shiffrin and Schneider, 1977; Neuman, 1984). *Executive attention*, in contrast, refers to attention processing related to responding and task execution. Research on selective attention relies on a range of tasks that are known as selective attention tasks. However, rarely ever a task is process-pure and, in fact, many of the selective attention tasks also involve executive attention processes, as will become clear later in this article. In the literature, a range of terms, such as *executive control, execution function*, and *cognitive control* has been used interchangeably to refer to the kind of attention involved in control of action. In the present article no strict distinction will be made between these terms.

## Working memory: executive attention and control

The concept of WM finds its origin in the context of research on short-term memory. Originally, the short-term memory system (STS) was believed to temporarily maintain incoming information before it is transferred to the (episodic) long-term memory system. These ideas were expressed in a range of short-term memory models published in the 1960s and 1970s; the most representative of these models was the so-called “modal model” proposed by Atkinson and Shiffrin (1968). Although in this conception the STS was essentially a (passive) store, it was also endowed with so-called control processes, such as coding and rehearsal. These control processes allow active restructuring of the information with the aim of increasing the memory system’s efficiency, and presumably require attention. Later on, the idea that the STS can also be used to temporarily maintain information in the service of other activities gradually gained ground. If control processes like rehearsal, coding, etc., are useful to improve long-term recall, these processes should also help short-term recall, as in looking up a phone number in the directory to make an immediate call (without the intention of remembering the phone number an hour or a day later). But no doubt, temporary storage of a piece of information can also be useful in other tasks, such as carrying or borrowing in mental arithmetic (e.g., Hitch, 1978; Imbo et al., 2007), or to maintain the premises into focus to reach a conclusion (e.g., Vandierendonck and De Vooght, 1997).

In fact, the consideration that short-term memory storage can be at the service of other tasks required a new approach to the study of temporary memory. For the study of STS, an examination of its performance in free recall was a suitable methodology. In contrast, an investigation of the properties of a memory system that provides services to other tasks, required a methodology in which the limiting capacities of the memory system can be measured in situations requiring different amounts of temporary storage in the service of another task. This resulted in two methodological changes. First, instead of free recall, serial recall became the standard method to measure the limits of the memory system because it is less vulnerable to familiarity-based recollections. Second, in order to examine memory-consumption by another task, a dual-task methodology was required. In such a methodology, typically a memory task is performed concurrently with another cognitive task that allegedly requires memory for its execution. On the basis of these methodological innovations, Baddeley and Hitch (1974) showed that the WM system had to provide flexible storage facilities supervised by a controlling agent; for this supervision they borrowed the notion of central executive from artificial intelligence research.

The framework introduced by Baddeley and Hitch (1974) can best be qualified as a multi-component WM. It originally consisted of two modality-specific slave systems (phonological loop and visuospatial WM) supervised by the central executive. This framework stimulated a large amount of research on WM. Apart from a range of new models on the storage facilities provided by the WM system (e.g., Logie, 1986, 1995; Hulme et al., 1991; Page and Norris, 1998, 2009; Burgess and Hitch, 1999, 2006; Brown et al., 2000), an approach to measure individual differences in WM capacity showed the importance of WM in processes such as reading (Daneman and Carpenter, 1980, 1983), counting (Case et al., 1982), mental arithmetic (Turner and Engle, 1989), and the relation of WM to intelligence (Engle et al., 1999). As research progressed and the number of dual-task studies increased, the call for fractionation of the central executive sounded louder (Baddeley, 1996a,b) and attempts to redefine this agent as a collection of executive functions (Miyake et al., 2000) or to recast the executive in terms of more basic executive processes (e.g., Szmalec et al., 2005; Vandierendonck et al., 2007, 2008) were published.

In all these studies, the role of attention was central. However, of the different attention networks distinguished by Posner (e.g., Posner and Petersen, 1990; Posner and Rothbart, 2007; Petersen and Posner, 2012) only the executive network is included in most present-day WM theories. This is completely consistent with the position that the role of the central executive corresponds with the supervisory attention model of Norman and Shallice (1986), which also basically requires the executive attention network to control actions. The question may be raised whether the WM system also calls on one or more of the other attention networks. In what follows, the utility of broadening the attention scope of theories of WM is further investigated. First, the breadth of the executive attention basis of WM theories is explored by a review of different attention tasks that are modulated by WM capacity. Next, attentional selectivity or orienting attention is considered, by briefly reviewing the evidence. Finally, an attempt is presented to integrate all these findings in a comprehensive view of the attentional basis of WM.

## Attention and working memory

To test whether WM includes particular forms of attention, basically two methodologies can be used. On the one hand, it is possible to use the traditional dual-task method in which two tasks calling on a particular resource are performed concurrently. When performance on either or both tasks is impaired compared to a single-task execution of these tasks, it follows that they are competing for this particular resource. In contrast, when two tasks allegedly tax different resources, concurrent performance of these tasks is not expected to result in performance impairments. Thus, a double dissociation can be established (e.g., Klauer and Zhao, 2004). On the other hand, an individual differences approach can be applied by selecting a group of participants with a high and a group with a low working-memory capacity (often top and bottom 25% of the distribution) as measured by one of the many dedicated WM span tasks (e.g., Daneman and Carpenter, 1980, 1983; Turner and Engle, 1989). If the factor of WM capacity interacts with a difficulty variation on the other task in such a way that the low capacity group’s performance suffers more from the difficulty variation than that of the high capacity group, it follows that the second task requires more WM capacity.

In the present section, the focus is on a range of attention tasks that require orienting towards or selection of particular stimuli that also require participants to ignore irrelevant or previously relevant stimuli. These tasks are known to involve controlled attention. As this term is sometimes used as a synonym for executive control, it seems quite likely that these tasks call on WM or tax some common resources or processes. In what follows, mostly behavioral studies are considered, although occasionally ERP findings are discussed as well. A useful review of electrophysiological studies of the relationship between selective attention and WM can be found in Gazzaley and Nobre (2012).

### Stroop task

Many attentional tasks require some form of control for their execution. Consider, for example, the Stroop (1935) task. In the standard form of the task, participants are shown words and are requested to name the color of the print. In incongruent trials, color words are presented shown in a color incongruent with the word meaning. Congruent trials consist of words in which the print color and the word name match. Sometimes, also neutral trials are shown in which the print color of a non-color word has to be named. In order to produce a correct answer, the relevant feature (print color) must be selected. In incongruent trials this is difficult because the irrelevant feature (the word meaning) is accessed automatically. The ensuing conflict must be resolved, which leads to slower and more error-prone responding. More particularly, the responses are slower than on congruent and neutral trials. Usually congruent and incongruent trials are mixed, and typically incongruent trials are slower when they are less frequent (MacLeod, 1991).

Several studies have shown that low-span participants show a larger Stroop interference effect (i.e., slower and more error-prone responding to incongruent than to congruent and neutral trials) than the high-span participants (Long and Prat, 2002; Kane and Engle, 2003; Kiefer et al., 2005; Meier and Kane, 2013). This difference is also modulated by the frequency of incongruent trials and the order in which blocks with few and many incongruent trials are presented. This is taken as evidence that high-span subjects are better able to keep the task goal active in WM (Kane and Engle, 2003; Morey et al., 2012).

In a series of experiments, Kim et al. (2005) varied the modality of the WM load. Thus they observed increased interference when the WM load and Stroop task were in the same modality (e.g., both verbal), no interference effect when the WM load was in a modality different from the Stroop task (e.g., verbal Stroop task with visuospatial WM load), and decreased interference when the WM load was in the same modality as the distracter of the Stroop task (e.g., both verbal). Other studies focused on modulation of post-conflict control. A study by Soutschek et al. (2013), for example, shows that a concurrent WM load modulates the post-conflict control. Over three experiments, different types of WM load were applied. When the WM task was an arithmetic updating task or an n-back task, but not when the WM task was a simple load task (recall a number of digits), the interaction of current trial congruency by previous trial congruency, which is a marker of post-conflict adaptation (Botvinick et al., 2001), was modulated by the WM load. In other words, the requirement to update WM contents depletes WM attentional resources to such an extent that it is no longer possible to perform control adjustments after an incongruent Stroop trial; simply maintaining a series of up to six digits does not have this effect.

### Flanker task

In the flanker task (Eriksen and Eriksen, 1974), participants are requested to categorize a central stimulus with a left or right key-press, while it is flanked by either compatible or incompatible stimuli. As an example, consider a central stimulus (left or right arrow) flanked by two stimuli on the left and two on the right; the flankers are also arrows, either all left pointing or all right pointing. When the flankers are compatible with the central stimulus (e.g., arrows pointing in the same direction), responses are faster than when the flankers are incompatible (Flanker Compatibility Effect, FCE). When stimulus and flankers are compatible they all favor the same response, but when they are incompatible they favor conflicting responses resulting in a slower response and a larger likelihood of an error. As in the Stroop task, also in the Flanker task, post-conflict adjustment has been observed (Botvinick et al., 2001).

Lavie et al. (2004) showed in a series of experiments that the FCE was more increased under a larger WM load. Pratt et al. (2011) compared flanker performance on an arrow-flanker task under single-task and dual-task conditions while recording early and late attention-sensitive event-related potentials (P1 and P300). In the dual-task condition, a memory load of 4 or 7 items (Sternberg task; Sternberg, 1966) was presented for later recall and during the retention interval a number of flanker trials were presented. The FCE was observed, and it was reduced under both load conditions. P300 amplitude to incompatible trials was also reduced under dual-task conditions. These findings suggest that under WM load it was more difficult to suppress interference from the incompatible flankers. The observation that P1 amplitude was reduced on all dual-task flanker (compatible and incompatible) trials showed that increased WM demands reduce top-down attentional control over early visual processing. A general FCE was also confirmed in a correlational study with structural equation modeling (Keye et al., 2009). This study also tested the role of WM in post-conflict adjustment, but could not confirm this role.

### Negative priming

When the presently relevant stimulus was present but irrelevant on the previous trial, it is said that the present stimulus is negatively primed. This results in a slower response to the relevant stimulus compared to a neutral situation where the stimulus was not present on the previous trial (Tipper, 1985; Tipper and Driver, 1988). Note that negative priming is the opposite of repetition priming where the previous and the current relevant stimulus are the same. Agreement about the mechanism behind negative priming is still lacking, but the competition between representations or processes linked to the previous (ignored event) and the present (relevant) event is part of most accounts. For that reason, it is likely that WM modulates negative priming.

This was confirmed in a study with negative priming in a letter-naming task under a range of conditions that varied the WM load from 0 to 4 words that were presented for later recall (Engle et al., 1995). Under loads 0–2, negative priming was present, but it became gradually smaller and disappeared completely from load 3 on. Because both the negative priming task and the WM load were verbal, it is possible that this result is due to a domain-specific interference. This was tested in another study that included both verbal and visuospatial WM loads (Conway et al., 1999). Two experiments used letter naming to investigate negative priming, combined with a WM load of 0–4 words in the first experiment and visuospatial WM load of 0–4 polygons in the second experiment. In addition, the participants were classified as low or high WM span on the basis of the operation span (OSPAN; Turner and Engle, 1989). Both experiments revealed the presence of negative priming, but this effect was only significant at load 0, irrespective of the type of WM load. It was also expected that the high-span participants would show more negative priming than the low-spans. The rationale for this expectation is that negative priming is the result of coping with interference and that high-span subjects are better able to handle interference. This expectation was also confirmed in the observation that only high-spans showed a negative priming effect at load 0, whereas low-spans showed no negative priming effect at all.

Long and Prat (2002) reported similar findings. One of their experiments concerning color word Stroop stimuli has been mentioned in the section of Stroop Tasks (above). In another experiment, they also used Stroop stimuli and compared neutral trials with incongruent trials, half of which were conflict trials (incongruent color word pairs) and the other half were negative priming trials where the previously ignored word was the color name of the present trial. In high-span participants, no naming latency difference was observed between neutral trials and conflict trials; this confirms the earlier reported findings about the Stroop interference effect. Negative priming (the RT difference between conflict trials and negative priming trials) was very high. In contrast, low spans showed a large Stroop interference effect and a small, but reliable negative priming effect. These findings corroborate earlier findings about Stroop interference and they are consistent with the findings reported by Conway and colleagues regarding negative priming (Engle et al., 1995; Conway et al., 1999).

It should also be noted that in contrast to identity-based negative priming as in all these reviewed studies, location-based negative priming is not affected by either a visuospatial or a verbal WM load (Kahan et al., 2013). Unfortunately, this study is not very convincing because the so-called memory load (of three items) was presented at a rate of 350 ms per item (300 ms on, 50 ms off), and the memory test did not require to recall or to recognize any of the items specifically; instead a judgment of frequency was asked (more even or odd numbers for the verbal load e.g.). The finding that such a load does not remove location-based negative priming does not seem to allow strong statements about the role of WM in location-based priming.

### Attentional blink

The attentional blink refers to an impaired ability to detect a second target during an interval of about 400–600 ms after detecting a first target (Raymond et al., 1992). The typical procedure for detecting the attentional blink consists of a rapid serial visual presentation (RSVP) of letters in which a letter is shown every 100 ms (the exact value varies slightly over studies). One letter is in a different color (e.g., white instead of black) and occurs as the first target (T1); on part of the trials a second target (e.g., X; T2) occurs in the same color as all the other letters and at the end of the series (usually about 20 letters), the question whether T2 was or was not present is to be answered. T2 is presented at various positions after T1. When presented on positions 2–7 after T1, the frequency of detecting T2 decreases; when presented immediately after T1, detection frequency is not impaired. There is a large literature on the attentional blink and many of the task parameters have been varied (rate of presentation, usage of additional targets, visual and verbal memory loads, etc.), sometimes leading to surprising outcomes.

Some studies have used a WM load while performing the RSVP-attentional blink task. The findings of these studies are somewhat variable, but some studies found no variation in the size of the attentional blink effect with increased memory load, although the memory load affected some performance aspects (e.g., Akyürek and Hommel, 2005, 2006). However, when participants had to judge whether T1 is part of the WM load, the attentional blink increased (Akyürek et al., 2007). Other studies measured WM capacity by means of the OSPAN and found that the attentional blink was decreased with higher WM capacity (Arnell et al., 2010) and that this was even the case when Raven’s standard progressive matrices scores were partialed out (Colzato et al., 2007).

### Stimulus-response compatibility

When stimulus features or dimensions overlap with response features or dimensions, stimulus-response compatibility (S-R compatibility) is bound to occur. Two types of S-R compatibility (see Kornblum et al., 1990) are of primary interest here, namely compatibility due to an overlap between the relevant stimulus and response dimensions (e.g., respond left to a left positioned or left-pointing stimulus) which is also known as S-R compatibility proper, and compatibility due to an overlap between an irrelevant stimulus dimension and the relevant response dimension. The Simon effect (e.g., Simon and Rudell, 1967) is an example of the latter: consider the request to respond with a left key-press to a red circle and to respond right to a green circle, responses will be faster if the red circle is positioned on the left side of the screen compared to when it is positioned on the right. Position on the screen is here irrelevant, but it affects responding. Both types of compatibility require action control, which is one of the typical expressions of executive control. Performance on such S-R compatibility tasks is therefore expected to be related to WM capacity or WM load. A few published studies are relevant to this issue, most of them concern the Simon effect.

There is a lot of variability in the methodologies used in these studies, which makes it difficult to extract a clear pattern of findings. Some studies report no or only a modest effect of a memory load on the Simon effect (Stins et al., 2004; Stürmer et al., 2005), whereas other studies found some effects (Zhao et al., 2010; Wühr and Biebl, 2011). It seems quite likely that the Simon effect is not very susceptible to WM load, especially as it seems rather easy to reverse the Simon effect (Notebaert et al., 2006). It is probably more interesting to follow the logic applied in studies of the Stroop effect and the FCE, and to look at conflict adaptation. Weldon et al. (2013) measured WM capacity in a Simon experiment. WM capacity was not related to performance on the Simon task, but a measure of the magnitude of the trial-by-trial conflict adaptation correlated negatively with WM capacity for low-span and near 0 for high-span participants.

### Interim conclusion

In this section, attention tasks were considered that involve both selection and control. A common theme among these tasks and the way they are performed is that in the selection of the relevant stimulus feature and consequently in performing the correct response, some form of conflict or competition between processes occurs that may cause erroneous and/or delayed responses. This is the case for the Stroop interference effect, the flanker compatibility effect, and the Simon effect. Incongruent or incompatible trials in each of these are based on a competition between irrelevant and relevant stimulus features or dimensions. In a particular respect, negative priming is similar, because a previously irrelevant stimulus becomes now relevant and as a consequence the action coupled to the stimulus has to be changed, creating a conflict between the old and the new action link. Only the attentional blink seems to be different, but it may be too early to draw conclusions on the underlying processes. In all these cases, attentional control is needed, and the evidence shows that the observed interference and its control (sometimes in a trial-by-trial conflict adjustment) is modulated by the individual’s amount of WM capacity and that increases of WM load modulate the observed effects. As a temporary conclusion, it may be said that all these forms of attention are mediated by WM or are at least calling on processes that are shared with WM.

## Working memory and visual search

In the present section, the focus is on selective attention tasks of which it is not clear that they involve executive attention. In particular, some forms of perceptual selectivity will be considered, such as attentional capture, visual (perceptual) search and environmental monitoring. In all these tasks, participants are given the instruction to search for a particular target. Usually this target is only briefly presented or described before the start of the search; therefore, it must be assumed that the searched-for object is active in WM.

### Attentional capture

Sometimes particular events stand out and capture attention so-to-speak automatically. For example, a single poppy in a lawn will be noticed immediately. Hence, searching for a singleton (stimulus with unique features) is rather easy, such as finding a red circle among green circles and squares. However, if the object of the search is to find the green square among green circles and one single red circle (irrelevant singleton), finding the target object may be hampered by the presence of the irrelevant singleton. The question is now considered whether such searches are mediated by WM.

Lavie and de Fockert (2005) used a search task where nine figures (circles and diamonds) were arranged in a circular lay out. All the figures were shown in red on a black background, except for an irrelevant color singleton (green circle) that was present on some trials. Each figure contained either a horizontal or a tilted line. The stimuli were presented for 200 ms and the requirement was to find the red circle among the red diamonds (and occasional green circle) and to decide on the orientation of the line. This task was performed either alone or in a dual-task situation with a verbal WM load (six digits). Search was slowed by the presence of the singleton, and this effect was augmented under load. This observation was further corroborated in an event-related fMRI study that showed that the presence of the singleton was associated with higher superior parietal activation (in line with a capture account) and higher frontal activity (Lavie and de Fockert, 2006). Behavioral singleton interference correlated with the frontal activity, and singleton interference was also higher under WM load.

Further specifications of the relationship between WM and attentional capture come from studies that used other types of WM load. One study used only a visual WM load and confirmed the finding that the presence of the WM load increased the singleton interference (Olivers et al., 2006). This study also observed that the effect was stronger when the irrelevant singleton overlapped with the WM load but only when difficult to verbalize pictures were used. A study of Burnham et al. (2014) explicitly tested the role of different WM components in attentional capture. They found that only tasks that tax visuospatial WM and executive control increased distracter interference, while a phonological WM load did not affect capture.

### Visual search

It thus seems that in at least some visual search tasks, a WM load modulates performance. Can these observations be generalized to other types of visual or perceptual search? A study by Kane et al. (2006) examined the relationship between WM capacity and visual search. In three experiments the investigators selected high and low complex span participants to perform a series of visual search tasks in which speed of response and search errors were registered. In the first experiment, participants searched a letter F among distracters (efficient search with letters O as distracter; inefficient search with letters E as distracter); set size, presence of the target, and organization of the layout were varied. Although strong and reliable search RT slopes were observed, WM span did not affect performance. In the second experiment, a similar design was used in which participants searched either for a red vertical line among green vertical lines and red and green horizontal lines (conjunction search) or for an F among E and 90° tilted T’s (spatial configuration search) in different set sizes. Again, reliable RT slopes were observed, but WM span did not affect or modulate the findings. In the final experiment, participants had to detect an F (regular or mirrored) among regular and mirrored E’s and T’s tilted 90° forward or backward. All the symbols were presented on three concentric rings at eight equally spread positions over the rings. Search was to be performed in two different ways. In one condition, the participants were requested to perform a search of the middle ring starting at the top (12 o’clock) position and following the positions clockwise until they found the first F (regular or mirrored; there could be more than one F). In the other condition, search was not constrained but was aimed to find the F on the middle ring (there was only one F on this ring). The constrained search (command search) was used because it had been reported that such search requires volition and is much slower than standard search (Wolfe et al., 2000). The command search was indeed slower than the unconstrained search, but again WM span did not affect neither modulate performance. These findings led Kane et al. (2006) to conclude that the executive control function of WM does not “generalize to difficult attention tasks lacking the need to actively maintain goals to restrain prepotent responses or constrain attentional focus to particular stimuli or locations in space amid distractors” (p. 771).

Nevertheless, it is important not to overstate the scope of these findings. Indeed, the already reported attentional capture studies show that search performance is affected by a WM load, in a task that is not dramatically different from the ones used by Kane et al. (2006). These authors themselves, moreover, remark that several dual-task studies did report effects of WM load on visual attention tasks (e.g., Woodman and Luck, 2004). Anderson et al. (2008) report a dual-task study of efficient and inefficient search performance. Participants were presented 4 or 10 randomly rotated L’s in a circular arrangement with the request to decide whether a target X (efficient search) or T (inefficient search) was present or not. The search task was performed in isolation or within the retention interval of a WM task with either a low (3 items) or a high load (5 items). Inefficient search, but not efficient search, was affected by the size of the memory load. This was the case for a spatial WM load as well as for a verbal WM load. These findings clearly show that at least inefficient visual search calls on domain-general WM resources. Given that this study used a task that is quite similar to one of the tasks used by Kane et al. the possibility that the correlational methodology used by these authors may be less sensitive to detecting WM modulation in visual search.

Findings like these may strengthen the impression that the methodology used (correlational or dual-task) plays an important role. No doubt, there are important differences between these methodologies (e.g., Logie, 2011), and the possibility that the correlational methodology used by these authors may be less sensitive to detecting WM modulation in visual search should not be rejected on *a priori* grounds. Yet, small changes to the design may result in different findings. Sobel et al. (2007) made some changes to the conjunction search task used by Kane et al. (2006) in order to allow a distinction between bottom-up and top-down search mechanisms. They found that searches based on bottom-up processes were not related to WM capacity, but searches based on top-down processes were performed better by high-span than by low-span participants.

That small changes to the design may indeed affect the results was also shown in a more recent study of Poole and Kane (2009). They presented target location cues for 1–8 target positions either followed by a long (1500 ms) or a short (30 ms) interval before the (inefficient) search display was shown. They found that high-span participants identified targets (F or mirrored F) faster than low-span subjects, but only when distracters were present on non-target positions, and only with long cue-stimulus intervals. Thus it seems that individual differences in visual search performance are only related to individual differences in WM capacity when it is necessary to maintain the search focus over a longer period and when distracters at non-focused positions are present.

### Input monitoring

Another aspect of search behavior is found *in situ*ations where the environment is monitored or scanned for the occurrence of a particular event, this is also known as input monitoring. On the basis of a conceptual analysis, Vandierendonck (2000a,b) proposed that input monitoring could be one of the more basic processes underlying executive control. In order to test the role of input monitoring, it was assumed that events occurring randomly distributed over time required more input monitoring effort than events occurring in a fixed time schedule. The rationale for this was that a fixed time schedule may be handled by automatic processes, while for randomly occurring events the monitoring process must be continuously adapted. Deschuyteneer and Vandierendonck (2005) investigated mental arithmetic performance (simple sums) while concurrently and continuously another task had to be performed that varied the degree of input monitoring and the involvement of response selection. These two variations were crossed. The secondary task consisted of high or low tones that were presented at a fixed tempo (1 tone every 1200 ms) or in an unpredictable tempo (random alternation of 900 and 1500 ms). Each tone required a response. In the simple response condition, one single response was to be emitted as soon as a tone was presented; in the response selection condition, low and high tones were responded to each with their associated response. The answer time to the arithmetic sums was slowed when a response selection was required compared to the requirement to produce a simple response. In contrast, answer times were not significantly different for fixed and random schedules of tone presentation, indicating that input monitoring is not part of the attentional resources required to execute the arithmetic sums. As it has been shown before that such sums call on WM (Hitch, 1978; Lemaire et al., 1996) and more specifically, on the executive control system (De Rammelaere et al., 1999, 2001; De Rammelaere and Vandierendonck, 2001; Imbo et al., 2005), these findings do not corroborate the hypothesis that input monitoring is part of executive control. In a similar study with calculation of arithmetic products as the primary task, these findings were confirmed: concurrent response selection but not concurrent input monitoring affected performance on the arithmetic task.

The hypothesis that input monitoring is part of executive control was also tested with saccades as primary task. Several studies have shown that anti-saccades (eye-movements away from a peripheral stimulus) but not pro-saccades (eye-movements towards a peripheral stimulus) call on WM’s executive system (e.g., Roberts et al., 1994; Stuyven et al., 2000; Kane et al., 2001). Vandierendonck et al. (2008) compared pro-saccade and anti-saccade execution either in a single-task condition or in a dual-task condition with a concurrent and continuous tone response task. There were four dual-task conditions resulting from orthogonal parametric variations in input monitoring and response selection (fixed vs. random tone intervals and simple or choice reaction task). Both pro- and anti-saccades suffered from a non-specific dual-task cost, but more interestingly, neither input monitoring nor response selection played any role in pro-saccades which are generally believed to be triggered automatically (Hallett, 1978; Kristjánsson et al., 2001), whereas anti-saccades were not only slower when response selection was required in the tone response task, but also when the spacing of the tones was random rather than fixed. The latter finding supports the hypothesis that input monitoring is part of the attentional control loop. It may be the case, though, that input monitoring overlaps more with eye-movement control than with executive control deployed in mental calculation.

Summarizing the results on input monitoring, it appears that input monitoring calls on executive attention when controlled saccades but not when automatic saccades have to be performed. However, arithmetic performance (simple sums and products) does not seem to be disturbed by an increased demand to monitor input. Note however, that these studies tested executive control without imposing a WM load. The present evidence therefore remains indirect and evidence directly involving WM operations is needed for a more solid support for the role of input monitoring in the attentional subsystem of WM.

## What links selective attention to executive control?

In balance, the evidence reviewed in the previous sections shows that in many cases selective attention tasks call on working memory, in particular on its executive attention control mechanism. However, in a number of situations selective attention operates without any executive demands (e.g., attentional capture, efficient visual search, …). The question that must be asked then is how working memory theory can account for these differences. In this section, the view is defended that selective attention taxes executive control depending on the characteristics of the task being executed. More specifically, the thesis will be developed that when a task (i.e., a goal-directed activity) is performed, a means-end representation is instantiated^1^ in working memory, also known as a task set, and when this representation includes attention selectivity as a means to achieve the task goal, and only then, task executive will call on executive demands. The view defended by Kim et al. (2005) with pools of modality-specific resources selectively contributing to content-based interference is not in contradiction with the present development as both views continue to build on Baddeley (1986) view on working memory.

The motivation for developing this view is the consideration that executive control as defined in some WM models (e.g., Baddeley and Hitch, 1974) still has the characteristics of a homunculus, notwithstanding the efforts that have been made to fractionate the central executive (Baddeley, 1996a,b, 2000; Miyake et al., 2000; Vandierendonck et al., 2007). The view developed here tries to specify the executive control processes in such a way that these processes are triggered whenever the appropriate conditions are met, so that no other supervisory control system is needed to overview proper application of these processes.

Taking into account that a range of studies did not find any effects of a WM load on task switching (e.g., Logan, 2004, 2006; Kane et al., 2007; Kiesel et al., 2007), while there is evidence showing that task switching calls on executive control processes (Goschke, 2000; Miyake et al., 2000; Baddeley et al., 2001; Friedman et al., 2008; Liefooghe et al., 2008), it seems that task and task-set representation are not competing with memory tasks for storage, but are competing for control processes. Yet, execution of a task requires a task set that remains active until the task is finished or abolished. According to some authors, whenever a task set becomes active, it is retrieved from long-term memory, and maintained into an active state in WM (e.g., Mayr and Kliegl, 2000). However, if the task set occupies WM storage space, it is expected that an increased WM load would impair task-switching performance and would do so even more when the task sets are more complex. Neither of these seems to be the case. Hence, if the task set is maintained in WM without affecting task-switching performance, it must be assumed that the task set is maintained separately from regular WM contents. A possible solution is to assume that the task set is maintained in a dedicated WM system for execution-related information, an *executive working memory module* (eWM), whereas regular WM storage is maintained in a kind of declarative WM module (dWM; Oberauer, 2009; Vandierendonck, 2012) or an episodic buffer (Baddeley, 2000) linking phonological and visuospatial representations to each other and to their long-term memory representations.

Before executing a new task, the intention to do so is adopted. This entails activation of a goal representation in WM and the retrieval of the task set from long-term memory. The goal representation is established by instantiating a label (e.g., the task name) referring to the goal or the task in dWM, and the task set is retrieved from long-term memory and configured in eWM. There are several reasons for making the assumption that dWM contains a reference to the goal. First, research has shown that other information present in dWM has an effect on task switching performance; more particularly, supportive information enhances performance while distracting information impairs performance in a task switching context (Goschke, 2000; Arrington, 2008). It may be argued that is at variance with findings that a WM load does not impair task-switching performance. However, in the context of task execution (also a memory task), a goal is always present and would thus always be part of dWM. Second, procedural knowledge that matches the contents of dWM, including the goal label, will be selected for execution in order to achieve the goal.

The task set is loaded in eWM. A task set is a collection of task-execution parameters that specify and constrain the actions that can be taken to achieve the task goal (cf. Logan and Gordon, 2001). For the rather simple cognitive tasks as the ones considered here (situations requiring solving of a new problem or solving of a complex problem are not considered here, because in such cases it cannot be assumed that long-term memory contains a complete task set as the means to attain the goal are not yet known), the task set will contain a representation of actions that lead to goal achievement. Other parameters that may be set during the instantiation of the task set include orientation of attention towards the relevant stimulus sets, setting a response threshold (determining speed/accuracy trade-off), maybe also a response bias, response modality, etc. (see also Vandierendonck et al., 2010). In this context, it is important to note that every intentional activity has a goal (represented in dWM) and a task set (stored in eWM). This implies that an intention to memorize some events for usage or recall some time later has also such representations.

Instead of presenting here a complete representation of the model (see Vandierendonck, 2012, for a more extensive description), a few examples will be elaborated to clarify the operation of the model. First, the operation of the model is described in performing a simple short-term memory task with immediate serial recall. Next, the model activity is described in performing a similar memory task with delayed recall, where the retention interval is filled with the execution of another cognitive task, as in dual-task situations as used in complex WM span tasks (Daneman and Carpenter, 1980; Turner and Engle, 1989) or in experiments parameterizing the amount of cognitive load (e.g., Barrouillet et al., 2004). Finally, it will be explained how the model does or does not call on selective attentional processing.

### Immediate serial recall

As recall is intended, a task goal for intentionally encoding and maintaining the sequence of events is instantiated in dWM, and the corresponding task set is configured in eWM. This is a rather simple task set, as there are no stimulus categorization rules or response categories. Task-set parameters will be needed to specify which subset of stimuli must be encoded, whether to use verbal rehearsal, or to use memory refreshing, whether chunking must be attempted and how big the chunks should be. Once the task set is configured, an automatic process continuously checks contents of dWM and eWM; when a content matches a condition-action rule in procedural LTM or one of the active task-set rules in eWM, the rule may be activated and the action specified in the rule is executed. Because this process continuously checks WM contents for execution of production rules, it is called the executive loop (a similar mechanism plays in production-rule models such as adaptive character of thought (ACT); Anderson and Lebiere, 1998). Each time a new memorandum is presented (e.g., a consonant), it will be encoded in dWM and a rehearsal or refreshment process will be applied to the new and to the previously presented memoranda. If necessary and suitable, chunking of memoranda in dWM may be attempted. This will create a new chunk, a new entity in long-term memory that contains the elements in their recorded order (which occasionally may be incorrect), or retrieve an already existing chunk from long-term memory. An instantiation of the chunk is added to dWM replacing the constituting elements. The executive loop continues until the recall signal occurs (or until another goal takes over). From this time on, the task goal changes from encode-and-maintain to maintain-and-recall. The goal representation in dWM is updated and the new task set is stored in eWM. The executive loop continues to run, but now the conditions have changed such that attention is no longer applied to incoming stimuli, but instead a retrieval loop searches dWM for the oldest element from the episode. When the oldest event is found and it is a chunk, an unpacking process is started that finds the oldest element within the chunk. Thus the stored elements are retrieved one by one and sent to the speech production process. It should be noted that due to the capacity limitation inherent to dWM, the number of memoranda that can be recalled in the correct order is limited to about 4–5 when no chunking is invoked; with chunking the number can be increased and may result in an average of 7–9 items (3–4 chunks).

### Serial recall with filled retention interval

Now a more complex task involving dual tasking is considered. In this example, a series of letters is presented for serial recall after a short retention interval at the end of presentation. During this retention interval, another task, namely parity judgment of digits must be executed: a series of digits are presented according to a paced schedule and for each digit a fast manual response is required categorizing the digit as odd (left key press) or even (right key press). During, the letter presentation part, basically the same series of events are taking place as in the previous example (encoding, rehearsing/refreshing, chunking the presented consonants), but because of the filled delay interval, at the end of presentation of the memoranda, a stimulus is presented that announces the requirement to perform another task. Up to that point, the encode-and-maintain goal is active. Next, dual tasking becomes necessary, which implies that two goals must be served, namely a goal to maintain the sequence of consonants (maintenance) and a goal to perform the instructed task (e.g., parity judgment) in response to the stimulus or stimuli (digits) presented during this retention period. What actually happens according to the model is that the presentation of a digit will (re)activate the parity goal and the task set for categorizing the digits as odd (left key-press) or even (right key-press). Other parameters that can be set at task configuration may depend on the specific instructions given at the start of the experiment (e.g., response threshold on the basis of requirements for speed and/or accuracy, the output modality, such as which hands and/or fingers to use, etc.). Each time a digit is presented (e.g., 4) it is instantiated in dWM. The executive loop will allow activation of the association “4-even”; this will result in adding “even” to dWM. Next, the rule “even-right” will fire, adding “right” to dWM. All these dWM events (“parity”, “4”, “even”, “right”) will be bound and result in application of the corresponding task-set rule, which results in producing a right key-press. The binding is now released and the elements (“parity”, “4”, “even”, “right”) are no longer needed and lose their activation. The parity goal is attained, and control shifts to the other goal (maintenance). This takes some time (goal-switching cost), but once the maintenance goal and task set are reactivated, the stored sequence is being rehearsed or refreshed. This goes until a next digit is presented which triggers reactivation of the parity goal and task set, or until the recall signal occurs. At recall, the same processes do occur as described in the immediate recall example.

Because the intervening activity uses storage facilities of dWM, on average, retrieval of the sequence of memoranda will become less efficient. If no chunking is attempted and hence only dWM is used for storage, this will most likely be the case. However, to the extent efforts were made during presentation of the memoranda to chunk them and outsource some of the storage to long-term memory, the bigger the likelihood that recall will not suffer much. Because similar series of memoranda and task executions follow each other, traces of previous task executions will keep some activity level in WM so as to create interference between present and past relevant elements. To the extent that such interference occurs, performance decrements will most likely occur. No doubt, occasionally such interference may also affect goal representations, leading to a more important performance failure.

### When does selective attention enter the picture?

Orientation of attention is encoded within the task set; it is one of the task-execution parameters/constraints. For example in configuring a task set for encoding and maintenance of information in an experimental context, the instructions usually specify where in the environment (e.g., center of the screen) which kind of memoranda (e.g., words, letters, etc.) will be presented. These instructions constitute ways to constrain the task set, which is encoded in the form of parameter settings. In other words, an intentional memorization task in principle always requires selective attention such as to encode the stimuli or stimulus aspects that must remembered and to ignore other ones. Therefore, if the participants comply with the instructions in the experiment, a WM load always includes selective attention.

Consider a few examples of a dual-task context. Suppose that there is a WM load and that during the retention interval a second task must be performed, for example, a visual search situation as was used by Anderson et al. (2008): during a WM load, either an efficient or an inefficient visual search must be performed. As already explained, a serial WM load engages selective attention in the memorization task set. The secondary task involves visual search. This requires a task set that also engages selective attention. Now, the search occurs in two variations. With efficient search (find X among L distracters), the X is so different from the distracters that it pops out. In fact, although the participant is prepared to selectively attend, the target is found automatically without effort and without in fact engaging controlled attention. In contrast, with inefficient search (find T among L), selective attention is needed and each element has to be checked whether it fits the target description. This involves control of the order in which positions are scanned and checking whether the target is present at that position. In comparison to efficient search, the action has to be controlled, and this also takes longer. This leaves less time to revert to the memory maintenance task to refresh the stored memoranda. For several reasons, then, it is to be expected that inefficient search interferes with WM whereas efficient search does not.

As another example, take a dual-task situation in which one task is intended to tax executive control, with less or more selective attention, but without memory load, and the other task involves less or more selective attention. In the review above, one such experiment compared performance on pro-saccade (eye movement towards a stimulus in peripheral view) and anti-saccade (eye movement away from peripheral stimulus) tasks, under a range of conditions meant to tax executive control processes (Vandierendonck et al., 2008). One contrast of interest is that between a task where an auditory stimulus either occurs at fixed intervals or occurs at variable intervals. This difference corresponds to situations with respectively low and high amounts of input monitoring. When the sounds occur at fixed intervals, a repetitive response can be programmed that more or less matches the stimulus rate. In contrast, when the sounds occur at random intervals, this is not possible and the aural input has to be scanned for the occurrence of a sound to which a response can then be programmed. In the latter case, the task set will engage controlled selective attention. However, only when the other task also engages controlled attention, impairment is expected to occur. In other words, with a pro-saccade task which is known to be performed automatically without any need for control processes (Hallett, 1978; Kristjánsson et al., 2001), the requirement for less or more input monitoring will make no difference, but in combination with an anti-saccade task, the presence of a high level of input monitoring creates a situations in which two tasks compete for the same resource, and it is expected that a higher level of input monitoring will make a difference. This is exactly what was observed in this study.

### Potential limitations

It can be argued that the view expressed in this modeling reduces executive control to one single executive function, namely task-set shifting. The model indeed assumes that the relevant task set is represented in a dedicated part of the working memory system (eWM) and that this representation together with the contents of dWM triggers the processes that result in goal achievement. Does this mean that processes engaged by other executive functions such as updating, inhibition, and others (Burgess, 1997; Rabbitt, 1997; Miyake et al., 2000) are excluded by the present view? They aren’t. For example, it is not at all clear that updating really is an executive function. Some authors have claimed that updating is not itself an executive function, but is rather a task demand, i.e., a requirement imposed by the task to continuously keep memory contents up-to-date (e.g., Szmalec et al., 2011). If memory updating is in fact a task demand, there is no doubt that a task-set representation can serve all what is needed. It seems evident that when the task is changed, completed or abolished, the related WM contents are no longer maintained in dWM. Besides, if the task set itself is no longer needed it will also be released from eWM. In other words, task changes result in an updating of the memory contents. A similar argument can be made for the executive function of inhibition. When particular memory contents are not useful to task execution, there is no task set that supports these contents and if they conflict with task execution, an inhibition process will be applied. Rather than defending a view based on bundles of processes as expressed in executive functions that themselves may easily develop into ill-defined agents or even homunculi, the present view attributes control to processes that are triggered when particular conditions are met, such as the presence of particular contents in dWM, the presence of a particular task set in eWM, and a knowledge base in (procedural) long-term memory that contains the appropriate rules that connect the conditions to actions or processes.

### Similarities to other modeling attempts

The model presented here is not a completely unique effort. Building on the multicomponent WM model of Baddeley and et al. (e.g., Baddeley and Hitch, 1974; Baddeley, 2000), it borrowed the production-rule logic as used in the ACT model (Anderson and Lebiere, 1998). Like Baddeley’s episodic buffer, the declarative WM module’s function is not only concerned with maintaining information in an active state, it is also needed for binding some of the contents. The present modeling was also influenced by Barrouillet’s time-based resource sharing model (Barrouillet et al., 2004, 2007). Barrouillet’s model attributes impaired recall in dual-task situations to the fact that the central attentional resource has to be time-shared between memory refreshments and task execution. This sharing involves rapid switching of attention from memory to task and vice versa. In the present model, the dominant task set determines which activity or process can be deployed (e.g., memory refreshment vs. parity judgment, e.g.), and the longer the time spent on executing the parity task, the less opportunity is left over for memory refreshment. One difference with Barrouillet’s model is that the present model does not assume rapid switching, but rather assumes that there is a cost associated with switching between memory refreshment and execution of another task.

The distinction between declarative and executive WM modules is reminiscent of Oberauer (2009, 2010) distinction between declarative and procedural WM. There are a few important differences however between Oberauer’s procedural WM (pWM) module and the executive WM module in the present model. Whereas pWM is considered to be activated procedural LTM, and hence essentially contains one or more stimulus-response mappings, eWM is not the activated part of procedural LTM, but is instead an autonomous module containing task set information, including parameters specifying task execution. While in Oberauer’s view, dWM and pWM have the same structure and perform the same kind of actions be it on different contents, in the present view, there is no analogy in the operation of dWM and eWM; each module has its own function but their contents enter a collaborative process to achieve the present goal.

## General discussion

The evidence regarding a role for WM in selective attention was reviewed and showed that very often, but not always, a WM load impaired performance in selective attention tasks. Similarly, correlational studies showed a relationship between individual WM capacity and selective attention performance in most but not all selective attention tasks. A model was presented that provides a basis for distinguishing selective attention tasks that do and those that do not interfere with WM. Basically, this model accounts for this difference by assuming that interference is bound to occur when both tasks (the WM task and the attention task) are prepared for attentional selectivity on condition the selective attention does not occur automatically.

While it would certainly be interesting to know whether WM (and its executive control mechanism) interact with other attention contexts than selective attention, the present paper did not discuss the interactions of executive control with the alerting network (Posner and Petersen, 1990). One reason for not following up this link concerns the difficulty to design experiments that are not contaminated by the presence of arousal, because the alerting network is intricately linked to arousal phenomena, while executive control in a dual-task context calls on effort which is also intricately linked to arousal.

Another attention mechanism that recently has gained interest concerns self-control (Petersen and Posner, 2012). An important part of research in recent years has indeed been concerned with aspects of self-control, volition, and the like. As shown by some studies on task switching, the WM system may provide an excellent substrate for self-control in the form of the phonological loop (Goschke, 2000; Baddeley et al., 2001; Emerson and Miyake, 2003; Miyake et al., 2004; Bryck and Mayr, 2005; Demanet et al., 2010; Vandierendonck et al., 2012). That there are links between executive control and self-control is beyond doubt, but should be the subject of a separate study.

## Conflict of interest statement

The author declares that the research was conducted in the absence of any commercial or financial relationships that could be construed as a potential conflict of interest. The Guest Associate Editor Jean-Philippe van Dijck declares that, despite being affiliated to the same institution as author André Vandierendonck, the review process was handled objectively and no conflict of interest exists.

## Footnotes

^1^Although it is often assumed that working memory consists of activated long-term memory (e.g., Mayr and Kliegl, 2003; Oberauer, 2009), others conceive WM as a temporary working space that is linked to long-term memory but separate from it. In line with the latter conception, elements stored in WM are not simply activated; the present model assumes that an instance is encoded in WM that combines perceived and retrieved features from LTM into a single trace.

## References

[B1] Akyürek E. G. Hommel B. (2005). Short-term memory and the attentional blink: capacity versus content. Mem. Cognit. 33, 654–663 10.3758/bf03195332 16248330

[B2] Akyürek E. G. Hommel B. (2006). Memory operations in rapid serial visual presentation. Eur. J. Cogn. Psychol. 18, 520–536 10.1080/09541440500423160

[B3] Akyürek E. G. Hommel B. Jolicoeur P. (2007). Direct evidence for a role of working memory in the attentional blink. Mem. Cognit. 35, 621–627 10.3758/bf03193300 17848020

[B4] Anderson E. J. Mannan S. K. Rees G. Sumner P. Kennard C. (2008). A role for spatial and nonspatial working memory processes in visual search. Exp. Psychol. 55, 301–312 10.1027/1618-3169.55.5.301 25116297

[B5] Anderson J. R. Lebiere C. (1998). The Atomic Components of Thought. New York: Lawrence Erlbaum Associates

[B6] Arnell K. M. Stokes K. A. MacLean M. H. Gicante C. (2010). Executive control processes of working memory predict attentional blink magnitude over and above storage capacity. Psychol. Res. 74, 1–11 10.1007/s00426-008-0200-4 19084999

[B7] Arrington C. M. (2008). The effect of stimulus availability on task choice in voluntary task switching. Mem. Cognit. 36, 991–997 10.3758/mc.36.5.991 18630205

[B8] Atkinson R. C. Shiffrin R. M. (1968). “Human memory: a proposed system and its control processes,” in The Psychology of Learning and Motivation (Vol. 2), eds Spence K. W. Spence J. T. (New York: Academic Press), 89–195

[B9] Baddeley A. (1986). Working Memory. Oxford: Oxford University Press

[B10] Baddeley A. (1996a). Exploring the central executive. Q. J. Exp. Psychol. 49A, 5–28 10.1080/713755608

[B11] Baddeley A. (1996b). The fractionation of working memory. Proc. Natl. Acad. Sci. U S A 93, 13468–13472 10.1073/pnas.93.24.13468 8942958 PMC33632

[B12] Baddeley A. (2000). The episodic buffer: a new component of working memory? Trends Cogn. Sci. 4, 417–423 10.1016/s1364-6613(00)01538-2 11058819

[B13] Baddeley A. Chincotta D. Adlam A. (2001). Working memory and the control of action: evidence from task switching. J. Exp. Psychol. Gen. 130, 641–657 10.1037/0096-3445.130.4.641 11757873

[B14] Baddeley A. D. Hitch G. (1974). “Working memory,” in The Psychology of Learning and Motivation (Vol. 8), ed Bower G. H. (New York: Academic Press), 47–89

[B15] Barrouillet P. Bemardin S. Portrat S. Vergauwe E. Camos V. (2007). Time and cognitive load in working memory. J. Exp. Psychol. Learn. Mem. Cogn. 33, 570–585 10.1037/0278-7393.33.3.570 17470006

[B16] Barrouillet P. Bernardin S. Camos V. (2004). Time constraints and resource sharing in adults’ working memory spans. J. Exp. Psychol. Gen. 133, 83–100 10.1037/0096-3445.133.1.83 14979753

[B17] Botvinick M. M. Braver T. S. Barch D. M. Carter C. S. Cohen J. D. (2001). Conflict monitoring and cognitive control. Psychol. Rev. 108, 624–652 10.1037/0033-295X.108.3.624 11488380

[B18] Brown G. D. A. Preece T. Hulme C. (2000). Oscillator-based memory for serial order. Psychol. Rev. 107, 127–181 10.1037/0033-295x.107.1.127 10687405

[B19] Bryck R. L. Mayr U. (2005). On the role of verbalization during task set selection: switching or serial order control? Mem. Cognit. 33, 611–623 10.3758/bf03195328 16248326

[B20] Burgess N. Hitch G. J. (1999). Memory for serial order: a network model of the phonological loop and its timing. Psychol. Rev. 106, 551–581 10.1037/0033-295x.106.3.551

[B21] Burgess N. Hitch G. J. (2006). A revised model of short-term memory and long-term learning of verbal sequences. J. Mem. Lang. 55, 627–652 10.1016/j.jml.2006.08.005

[B22] Burgess P. W. (1997). “Theory and methodology in executive function research,” in Methodology of Frontal and Executive Function, ed Rabbitt P. (Hove: Psychology Press), 81–116

[B23] Burnham B. R. Sabia M. Langan C. (2014). Components of working memory and visual selective attention. J. Exp. Psychol. Hum. Percept. Perform. 40, 391–403 10.1037/a0033753 23875574

[B24] Case R. Kurland D. M. Goldberg J. (1982). Operational efficiency and the growth of short-term-memory span. J. Exp. Child Psychol. 33, 386–404 10.1016/0022-0965(82)90054-6

[B25] Colzato L. S. Spape M. M. A. Pannebakker M. M. Hommel B. (2007). Working memory and the attentional blink: blink size is predicted by individual differences in operation span. Psychon. Bull. Rev. 14, 1051–1057 10.3758/bf03193090 18229474

[B26] Conway A. R. A. Tuholski S. W. Shisler R. J. Engle R. W. (1999). The effect of memory load on negative priming: an individual differences investigation. Mem. Cognit. 27, 1042–1050 10.3758/bf03201233 10586579

[B27] Daneman M. Carpenter P. A. (1980). Individual differences in working memory. J. Verbal Learn. Verbal Behav. 19, 450–466 10.1016/S0022-5371(80)90312-6

[B28] Daneman M. Carpenter P. A. (1983). Individual differences in integrating information between and within sentences. J. Exp. Psychol. Learn. Mem. Cogn. 9, 561–584 10.1037/0278-7393.9.4.561

[B29] De Rammelaere S. Vandierendonck A. (2001). Are executive processes used to solve simple mental arithmetic production tasks? Curr. Psychol. Lett. 2, 79–89

[B30] De Rammelaere S. Stuyven E. Vandierendonck A. (1999). The contribution of working memory resources in the verification of simple mental arithmetic sums. Psychol. Res. 62, 72–77 10.1007/s004260050041

[B31] De Rammelaere S. Stuyven E. Vandierendonck A. (2001). Verifying simple arithmetic sums and products: are the phonological loop and the central executive involved? Mem. Cognit. 29, 267–273 10.3758/bf03194920 11352209

[B32] Demanet J. Verbruggen F. Liefooghe B. Vandierendonck A. (2010). Voluntary task switching under load: contribution of top-down and bottom-up factors in goal-directed behavior. Psychon. Bull. Rev. 17, 387–393 10.3758/PBR.17.3.387 20551363

[B33] Deschuyteneer M. Vandierendonck A. (2005). Are ‘input monitoring’ and ‘response selection’ involved in solving simple mental additions. Eur. J. Cogn. Psychol. 17, 343–370 10.1080/09541440440000032

[B34] Emerson M. J. Miyake A. (2003). The role of inner speech in task switching: a dual-task investigation. J. Mem. Lang. 48, 148–168 10.1016/s0749-596x(02)00511-9

[B35] Engle R. W. Conway A. R. A. Tuholski S. W. Shisler R. J. (1995). A resource account of inhibition. Psychol. Sci. 6, 122–125 10.1111/j.1467-9280.1995.tb00318.x

[B36] Engle R. W. Tuholski S. W. Laughlin J. E. Conway A. R. (1999). Working memory, short term memory and general fluid intelligence: a latent variable approach. J. Exp. Psychol. Gen. 128, 309–311 10.1037/0096-3445.128.3.309 10513398

[B37] Eriksen B. A. Eriksen C. W. (1974). Effects of noise letters upon the identification of a target in a non-search task. Percept. Psychophys. 16, 143–149 10.3758/bf03203267

[B38] Friedman N. P. Miyake A. Young S. E. DeFries J. C. Corley R. P. Hewitt J. K. (2008). Individual differences in executive functions are almost entirely genetic in origin. J. Exp. Psychol. Gen. 137, 201–225 10.1037/0096-3445.137.2.201 18473654 PMC2762790

[B39] Gazzaley A. Nobre A. C. (2012). Top-down modulation: bridging selective attention and working memory. Trends Cogn. Sci. 16, 129–135 10.1016/j.tics.2011.11.014 22209601 PMC3510782

[B40] Goschke T. (2000). “Intentional reconfiguration and involuntary persistence in task set switching,” in Control of Cognitive Processes: Attention and Performance XVIII, eds Monsell S. Driver J. S. (Cambridge, MA: MIT Press), 331–355

[B41] Hallett P. E. (1978). Primary and secondary saccades to goals defined by instructions. Vision Res. 18, 1279–1296 10.1016/0042-6989(78)90218-3 726270

[B42] Hitch G. (1978). The role of short-term working memory in mental arithmetic. Cogn. Psychol. 10, 302–323 10.1016/0010-0285(78)90002-6

[B43] Hulme C. Maughan S. Brown G. D. A. (1991). Memory for familiar and unfamiliar words: evidence for a long-term memory contribution to short-term memory span. J. Mem. Lang. 30, 685–701 10.1016/0749-596x(91)90032-f

[B44] Imbo I. De Rammelaere S. Vandierendonck A. (2005). New insights in the role of working memory in carry and borrow operations. Psychol. Belg. 45, 101–121 10.5334/pb-45-2-101

[B45] Imbo I. Vandierendonck A. De Rammelaere S. (2007). The role of working memory in the carry operation of mental arithmetic: number and value of the carry. Q. J. Exp. Psychol. (Hove) 60, 708–731 10.1080/17470210600762447 17455078

[B46] Kahan T. A. Oldak V. A. Lichtman A. S. (2013). Working memory loads affect location-based negative priming differently than inhibition of return. J. Cogn. Psychol. 25, 473–492 10.1080/20445911.2013.789855

[B47] Kane M. J. Bleckley M. K. Conway A. R. A. Engle R. W. (2001). A controlled-attention view of working-memory capacity. J. Exp. Psychol. Gen. 130, 169–183 10.1037/0096-3445.130.2.169 11409097

[B48] Kane M. J. Conway A. R. A. Hambrick D. Z. Engle R. W. (2007). “Variation in working memory capacity as variation in executive attention and control,” in Variations in Working Memory, eds Conway A. R. A. Jarrold C. Kane M. J. Miyake A. Towse J. N. (New York: Oxford University Press), 21–48

[B49] Kane M. J. Engle R. W. (2003). Working-memory capacity and the control of attention: the contributions of goal neglect, response competition and task set to stroop interference. J. Exp. Psychol. Gen. 132, 47–70 10.1037/0096-3445.132.1.47 12656297

[B50] Kane M. J. Poole B. J. Tuholski S. W. Engle R. W. (2006). Working memory capacity and the top-down control of visual search: exploring the boundaries of “executive attention”. J. Exp. Psychol. Learn. Mem. Cogn. 32, 749–777 10.1037/0278-7393.32.4.749 16822145

[B51] Keye D. Wilhelm O. Oberauer K. van Ravenzwaaij D. (2009). Individual differences in conflict-monitoring: testing means and covariance hypothesis about the Simon and the Eriksen Flanker task. Psychol. Res. 73, 762–776 10.1007/s00426-008-0188-9 19034502

[B52] Kiefer M. Ahlegian M. Spitzer M. (2005). Working memory capacity, indirect semantic priming and stroop interference: pattern of interindividual prefrontal performance differences in healthy volunteers. Neuropsychology 19, 332–344 10.1037/0894-4105.19.3.332 15910119

[B53] Kiesel A. Wendt M. Peters A. (2007). Task switching: on the origin of response congruency effects. Psychol. Res. 71, 117–125 10.1007/s00426-005-0004-8 16133574

[B54] Kim S. Y. Kim M. S. Chun M. M. (2005). Concurrent working memory load can reduce distraction. Proc. Natl. Acad. Sci. U S A 102, 16524–16529 10.1073/pnas.0505454102 16258067 PMC1283430

[B55] Klauer K. C. Zhao Z. M. (2004). Double dissociations in visual and spatial short-term memory. J. Exp. Psychol. Gen. 133, 355–381 10.1037/0096-3445.133.3.355 15355144

[B56] Kornblum S. Hasbroucq T. Osman A. (1990). Dimensional overlap: cognitive basis for stimulus-response compatibility—a model and taxonomy. Psychol. Rev. 97, 253–270 10.1037//0033-295x.97.2.253 2186425

[B57] Kristjánsson A. Chen Y. Nakayama K. (2001). Less attention is more in the preparation of antisaccades, but not prosaccades. Nat. Neurosci. 4, 1037–1042 10.1038/nn723 11547337

[B58] Lavie N. de Fockert J. (2005). The role of working memory in attentional capture. Psychon. Bull. Rev. 12, 669–674 10.3758/bf03196756 16447380

[B59] Lavie N. de Fockert J. (2006). Frontal control of attentional capture in visual search. Vis. Cogn. 14, 863–876 10.1080/13506280500195953

[B60] Lavie N. Hirst A. de Fockert J. W. Viding E. (2004). Load theory of selective attention and cognitive control. J. Exp. Psychol. Gen. 133, 339–354 10.1037/0096-3445.133.3.339 15355143

[B61] Lemaire P. Abdi H. Fayol M. (1996). The role of working memory resources in simple cognitive arithmetic. Eur. J. Cogn. Psychol. 8, 73–103 10.1080/095414496383211

[B62] Liefooghe B. Barrouillet P. Vandierendonck A. Camos V. (2008). Working memory costs of task switching. J. Exp. Psychol. Learn. Mem. Cogn. 34, 478–494 10.1037/0278-7393.34.3.478 18444750

[B63] Logan G. D. (2004). Working memory, task switching and executive control in the task span procedure. J. Exp. Psychol. Gen. 133, 218–236 10.1037/0096-3445.133.2.218 15149251

[B64] Logan G. D. (2006). Out with the old, in with the new: more valid measures of switch cost and retrieval time in the task span procedure. Psychon. Bull. Rev. 13, 139–144 10.3758/bf03193825 16724781

[B65] Logan G. D. Gordon R. D. (2001). Executive control of attention in dual-task situations. Psychol. Rev. 108, 393–434 10.1037/0033-295x.108.2.393 11381835

[B66] Logie R. H. (1986). Visuo-spatial processes in working memory. Q. J. Exp. Psychol. 38A, 229–247 10.1080/14640748608401596 3737975

[B67] Logie R. H. (1995). Visuo-Spatial Working Memory. Hillsdale, NJ: Lawrence Erlbaum Associates

[B68] Logie R. H. (2011). The functional organization and capacity limits of working memory. Curr. Dir. Psychol. Sci. 20, 240–245 10.1177/0963721411415340

[B69] Long D. L. Prat C. S. (2002). Working memory and stroop interference: an individual differences investigation. Mem. Cognit. 30, 294–301 10.3758/bf03195290 12035891

[B70] MacLeod C. M. (1991). Half a century of research on the stroop effect: an integrative review. Psychol. Bull. 109, 163–203 10.1037/0033-2909.109.2.163 2034749

[B71] Mayr U. Kliegl R. (2000). Task-set switching and long-term memory retrieval. J. Exp. Psychol. Learn. Mem. Cogn. 26, 1124–1140 10.1037//0278-7393.26.5.1124 11009248

[B72] Mayr U. Kliegl R. (2003). Differential effects of cue changes and task changes on task-set selection costs. J. Exp. Psychol. Learn. Mem. Cogn. 29, 362–372 10.1037/0278-7393.29.3.362 12776747

[B73] Meier M. E. Kane M. J. (2013). Working memory capacity and stroop interference: global versus local indices of executive control. J. Exp. Psychol. Learn. Mem. Cogn. 39, 748–759 10.1037/a0029200 22774858

[B74] Miyake A. Emerson M. J. Padilla F. Ahn J. C. (2004). Inner speech as a retrieval aid for task goals: the effects of cue type and articulatory suppression in the random task cuing paradigm. Acta Psychol. (Amst) 115, 123–142 10.1016/j.actpsy.2003.12.004 14962397

[B75] Miyake A. Friedman N. P. Emerson M. J. Witzki A. H. Howerter A. Wager T. D. (2000). The unity and diversity of executive functions and their contributions to complex “frontal lobe” tasks: a latent variable analysis. Cogn. Psychol. 41, 49–100 10.1006/cogp.1999.0734 10945922

[B76] Morey C. C. Elliott E. M. Wiggers J. Eaves S. D. Shelton J. T. Mall J. T. (2012). Goal-neglect links stroop interference with working memory capacity. Acta Psychol. (Amst) 141, 250–260 10.1016/j.actpsy.2012.05.013 22749714

[B77] Neuman O. (1984). “Automatic processing: a review of recent findings and a plea for an old theory,” in Cognition and Motor Processes, eds Prinz W. Sanders A. (Berlin: Springer), 255–293

[B78] Norman D. A. Shallice T. (1986). “Attention to action: willed and automatic control of behavior,” in Consciousness and Self-Regulation (Vol. 4), eds Davidson R. J. Schwarts G. E. Shapiro D. (New York: Plenum Press), 1–18

[B79] Notebaert W. Gevers W. Verguts T. Fias W. (2006). Shared spatial representations for numbers and space: the reversal of the snarc and the Simon effects. J. Exp. Psychol. Hum. Percept. Perform. 32, 1197–1207 10.1037/0096-1523.32.5.1197 17002531

[B80] Oberauer K. (2009). “Design for a working memory,” in Psychology of Learning and Motivation: Advances in Research and Theory (Vol. 51), ed Ross B. H. (San Diego: Elsevier Academic Press Inc.), 45–100

[B81] Oberauer K. (2010). Declarative and procedural working memory: common principles, common capacity limits? Psychol. Belg. 50, 277–308 10.5334/pb-50-3-4-277

[B82] Olivers C. N. L. Meijer F. Theeuwes J. (2006). Feature-based memory-driven attentional capture: visual working memory content affects visual attention. J. Exp. Psychol. Hum. Percept. Perform. 32, 1243–1265 10.1037/0096-1523.32.5.1243 17002535

[B83] Page M. P. A. Norris D. (1998). The primacy model: a new model of immediate serial recall. Psychol. Rev. 105, 761–781 10.1037/0033-295x.105.4.761-781 9830378

[B84] Page M. P. A. Norris D. (2009). A model linking immediate serial recall, the Hebb repetition effect and the learning of phonological word forms. Philos. Trans. R. Soc. Lond. B Biol. Sci. 364, 3737–3753 10.1098/rstb.2009.0173 19933143 PMC2846317

[B85] Petersen S. E. Posner M. I. (2012). The attention system of the human brain: 20 years after. Annu. Rev. Neurosci. 35, 73–89 10.1146/annurev-neuro-062111-150525 22524787 PMC3413263

[B86] Poole B. J. Kane M. J. (2009). Working-memory capacity predicts the executive control of visual search among distractors: the influences of sustained and selective attention. Q. J. Exp. Psychol. (Hove) 62, 1430–1454 10.1080/17470210802479329 19123118

[B87] Posner M. I. Petersen S. E. (1990). The attention system of the human brain. Annu. Rev. Neurosci. 13, 25–42 10.1146/annurev.neuro.13.1.25 2183676

[B88] Posner M. I. Rothbart M. K. (2007). Research on attention networks as a model for the integration of psychological science. Annu. Rev. Psychol. 58, 1–23 10.1146/annurev.psych.58.110405.085516 17029565

[B89] Pratt N. Willoughby A. Swick D. (2011). Effects of working memory load on visual selective attention: behavioral and electrophysiological evidence. Front. Hum. Neurosci. 5:57 10.3389/fnhum.2011.00057 21716633 PMC3115462

[B90] Rabbitt P. (1997). “Introduction: methodologies and models in the study of executive function,” in Methodology of Frontal and Executive Function, ed Rabbitt P. (Hove: Psychology Press), 1–38

[B91] Raymond J. E. Shapiro K. L. Arnell K. M. (1992). Temporary suppression of visual processing in an RSVP task: an attentional blink. J. Exp. Psychol. Hum. Percept. Perform. 18, 849–860 10.1037/0096-1523.18.3.849 1500880

[B92] Roberts R. J. Jr. Hager L. D. Heron C. (1994). Prefrontal cognitive processes: working memory and inhibition in the antisaccade task. J. Exp. Psychol. Gen. 123, 374–393 10.1037/0096-3445.123.4.374

[B93] Schneider W. Shiffrin R. M. (1977). Controlled and automatic information processing. I. Detection, search and attention. Psychol. Rev. 84, 1–66 10.1037/0033-295x.84.1.1

[B94] Shiffrin R. M. Schneider W. (1977). Controlled and automatic human information processing. II. Perceptual learning, autonomic attending and a general theory. Psychol. Rev. 84, 127–190 10.1037/0033-295x.84.2.127

[B95] Simon J. R. Rudell A. P. (1967). Auditory S-R compatibility: the effect of an irrelevant cue on information processing. J. Appl. Psychol. 51, 300–304 10.1037/h0020586 6045637

[B96] Sobel K. V. Gerrie M. P. Poole B. J. Kane M. J. (2007). Individual differences in working memory capacity and visual search: the roles of top-down and bottom-up processing. Psychon. Bull. Rev. 14, 840–845 10.3758/bf03194109 18087947

[B97] Soutschek A. Strobach T. Schubert T. (2013). Working memory demands modulate cognitive control in the stroop paradigm. Psychol. Res. 77, 333–347 10.1007/s00426-012-0429-9 22391935

[B98] Sternberg S. (1966). High speed scanning in human memory. Science 153, 652–654 10.1126/science.153.3736.652 5939936

[B99] Stins J. F. Vosse S. Boomsma D. I. de Geus E. J. C. (2004). On the role of working memory in response interference. Percept. Mot. Skills 99, 947–958 10.2466/pms.99.7.947-958 15648493

[B100] Stroop J. R. (1935). Studies of inteference in serial verbal reactions. J. Exp. Psychol. 18, 643–662 10.1037/h0054651

[B101] Stürmer B. Seiss E. Leuthold H. (2005). Executive control in the Simon task: a dual-task examination of response priming and its suppression. Eur. J. Cogn. Psychol. 17, 590–618 10.1080/09541440540000077

[B102] Stuyven E. Van der Goten K. Vandierendonck A. Claeys K. Crevits L. (2000). Saccadic eye movements under conditions of cognitive load. Acta Psychol. 104, 69–85 10.1016/S0001-6918(99)00054-2 10769940

[B103] Szmalec A. Vandierendonck A. Kemps E. (2005). Response selection involves executive control: evidence from the selective interference paradigm. Mem. Cognit. 33, 531–541 10.3758/bf03193069 16156187

[B104] Szmalec A. Verbruggen F. Vandierendonck A. Kemps E. (2011). Control of interference during working memory updating. J. Exp. Psychol. Hum. Percept. Perform. 37, 137–151 10.1037/a0020365 20731517

[B105] Tipper S. P. (1985). The negative priming effect: inhibitory priming by ignored objects. Q. J. Exp. Psychol. A 37A, 571–590 10.1080/14640748508400920 4081101

[B106] Tipper S. P. Driver J. (1988). Negative priming between pictures and words: evidence for semantic analysis of ignored stimuli. Mem. Cognit. 16, 64–70 10.3758/bf03197746 2448578

[B107] Turner M. L. Engle R. W. (1989). Is working memory capacity task dependent? J. Mem. Lang. 28, 127–154 10.1016/0749-596x(89)90040-5

[B108] Vandierendonck A. (2000a). Bias and processing capacity in generation of random time intervals. Cogn. Sci. Q. 1, 205–233 10.1016/S0001-6918(99)00054-2

[B109] Vandierendonck A. (2000b). Is judgment of random time intervals biased and capacity limited? Psychol. Res. 63, 199–209 10.1007/pl00008179 10946594

[B110] Vandierendonck A. (2012). Role of working memory in task switching. Psychol. Belg. 52, 229–253 10.5334/pb-52-2-3-229

[B111] Vandierendonck A. De Vooght G. (1997). Working memory constraints on linear reasoning with spatial and temporal contents. Q. J. Exp. Psychol. A 50A, 803–820 10.1080/027249897391892 9450380

[B112] Vandierendonck A. Demanet J. Liefooghe B. Verbruggen F. (2012). A chain-retrieval model for voluntary task switching. Cogn. Psychol. 65, 241–283 10.1016/j.cogpsych.2012.04.003 22609806

[B113] Vandierendonck A. Deschuyteneer M. Depoorter A. Drieghe D. (2008). Input monitoring and response selection as components of executive control in prosaccades and antisaccades. Psychol. Res. 72, 1–11 10.1007/s00426-006-0078-y 16924540

[B114] Vandierendonck A. Liefooghe B. Verbruggen G. (2010). Task switching: interplay of reconfiguration and interference control. Psychol. Bull. 136, 601–626 10.1037/a0019791 20565170

[B115] Vandierendonck A. Szmalec A. Deschuyteneer M. Depoorter A. (2007). “Towards a multicomponential view of executive control. The case of response selection,” in Working Memory: Behavioural and Neural Correlates, eds Osaka N. Logie R. (Oxford: Oxford University Press), 247–259

[B116] Weldon R. B. Mushlin H. Kim B. Sohn M. H. (2013). The effect of working memory capacity on conflict monitoring. Acta Psychol. (Amst) 142, 6–14 10.1016/j.actpsy.2012.10.002 23165200

[B117] Wolfe J. M. Alvarez G. A. Horowitz T. S. (2000). Attention is fast but volition is slow. Nature 406:691 10.1038/35021132 10963584

[B118] Woodman G. F. Luck S. J. (2004). Visual search is slowed when visuospatial working memory is occupied. Psychon. Bull. Rev. 11, 269–274 10.3758/bf03196569 15260192

[B119] Wühr P. Biebl R. (2011). The role of working memory in spatial S-R correspondence effects. J. Exp. Psychol. Hum. Percept. Perform. 37, 442–454 10.1037/a0020563 20822296

[B120] Zhao X. A. Chen A. T. West R. (2010). The influence of working memory load on the Simon effect. Psychon. Bull. Rev. 17, 687–692 10.3758/PBR.17.5.687 21037167

